# Transcriptomic responses to biotic stresses in Malus x domestica: a meta-analysis study

**DOI:** 10.1038/s41598-018-19348-4

**Published:** 2018-01-31

**Authors:** Bipin Balan, Francesco Paolo Marra, Tiziano Caruso, Federico Martinelli

**Affiliations:** Dipartimento di Scienze Agrarie e Forestali, Università degli Studi di Palermo, Palermo, Italy

## Abstract

RNA-Seq analysis is a strong tool to gain insight into the molecular responses to biotic stresses in plants. The objective of this work is to identify specific and common molecular responses between different transcriptomic data related to fungi, virus and bacteria attacks in Malus x domestica. We analyzed seven transcriptomic datasets in Malus x domestica divided in responses to fungal pathogens, virus (Apple Stem Grooving Virus) and bacteria (Erwinia amylovora). Data were dissected using an integrated approach of pathway- and gene- set enrichment analysis, Mapman visualization tool, gene ontology analysis and inferred protein-protein interaction network. Our meta-analysis revealed that the bacterial infection enhanced specifically genes involved in sugar alcohol metabolism. Brassinosteroids were upregulated by fungal pathogens while ethylene was highly affected by Erwinia amylovora. Gibberellins and jasmonates were strongly repressed by fungal and viral infections. The protein-protein interaction network highlighted the role of WRKYs in responses to the studied pathogens. In summary, our meta-analysis provides a better understanding of the Malus X domestica transcriptome responses to different biotic stress conditions; we anticipate that these insights will assist in the development of genetic resistance and acute therapeutic strategies. This work would be an example for next meta-analysis works aiming at identifying specific common molecular features linked with biotic stress responses in other specialty crops.

## Introduction

Apple (Malus x domestica Borkh) is one of the most important cultivated tree fruit crops in temperate climates. Unfortunately this crop is severely affected by diseases mainly caused by fungi^1–3^, bacteria^4^ and viruses^5^ with a consequent drastic reduction in fruit quantity and quality that threatens grower’s profit.

Transcriptomic studies are usually conducted in a singular time, they do not provide any repetition across different seasons and frequently they are performed in field conditions where environmental variability is high and disturbing factors are frequently present. The identification of up- or down-regulated genes is often not enough to draw meaningful biological conclusions because it is hard to identify which gene plays a key role in specific signaling networks in host responses^1^. This issue leads to high difficulties in deriving conclusive models for understanding disease symptomatology. For these reasons more meta-analysis are needed in order to validate singular transcriptomic works with other similar studies performed with same research purposes. Meta-analysis of transcriptomic data will identify commonalities and differences between differentially regulated gene lists and will allow screen which genes are key players in gene-gene and protein-protein interaction networks. These analyses will allow delivering important information on how a specific environmental factor affects plant molecular responses and how plants activate general stress responses to environmental stresses^6^. An early “stress condition” in plants is similar to the “inflammatory response” occurring in animals in response to pathogen-associated factors. The identification of common genes between different biotic stress will allow to gain insight into these general responses and help the diagnosis of an early “stress state” of the plants. These analyses help in monitoring stressed plants to start early specific management procedures for each disease or disorder. The activation of common responses to different biotic stresses may precede the onset of symptoms, where more physiological changes lead to specific phenotypic changes and peculiar metabolic dysfunctions^7,8^. Indeed there is a strong need of compelling cases in order to generalize results across studies performed in the same crop and determine the most reliable and meaningful information linked with agronomic factors such as biotic stress responses.

In this work we have performed a meta-analysis of all transcriptomic data related to biotic stresses in Malus x domestica available at the moment. We aimed to determine which genes, pathways, gene set categories and predicted protein-protein interaction networks may play key roles in specific responses to pathogen infections.

## Results

### Meta-analysis of transcriptome data

We determined the list of the up- and down-regulated genes and we compared these lists in order to identify common and different regulated genes between the 7 studied research works dealing with biotic stress responses. We have normalized the data using the same log Fold change and p-values (log2 FC > 1 or log2 FC < −1; p-value < 0.05)(Supplementary Table 1).Venn diagrams showed the numbers of specific and commonly regulated genes between the three types of biotic stresses in Malus (Supplementary Figure 1). We found that 16 genes were commonly regulated in responses to fungal pathogens, virus (Apple Stem Grooving Virus) and bacteria (Erwinia amylovora).

### Gene ontology analysis

**Figure 1:**
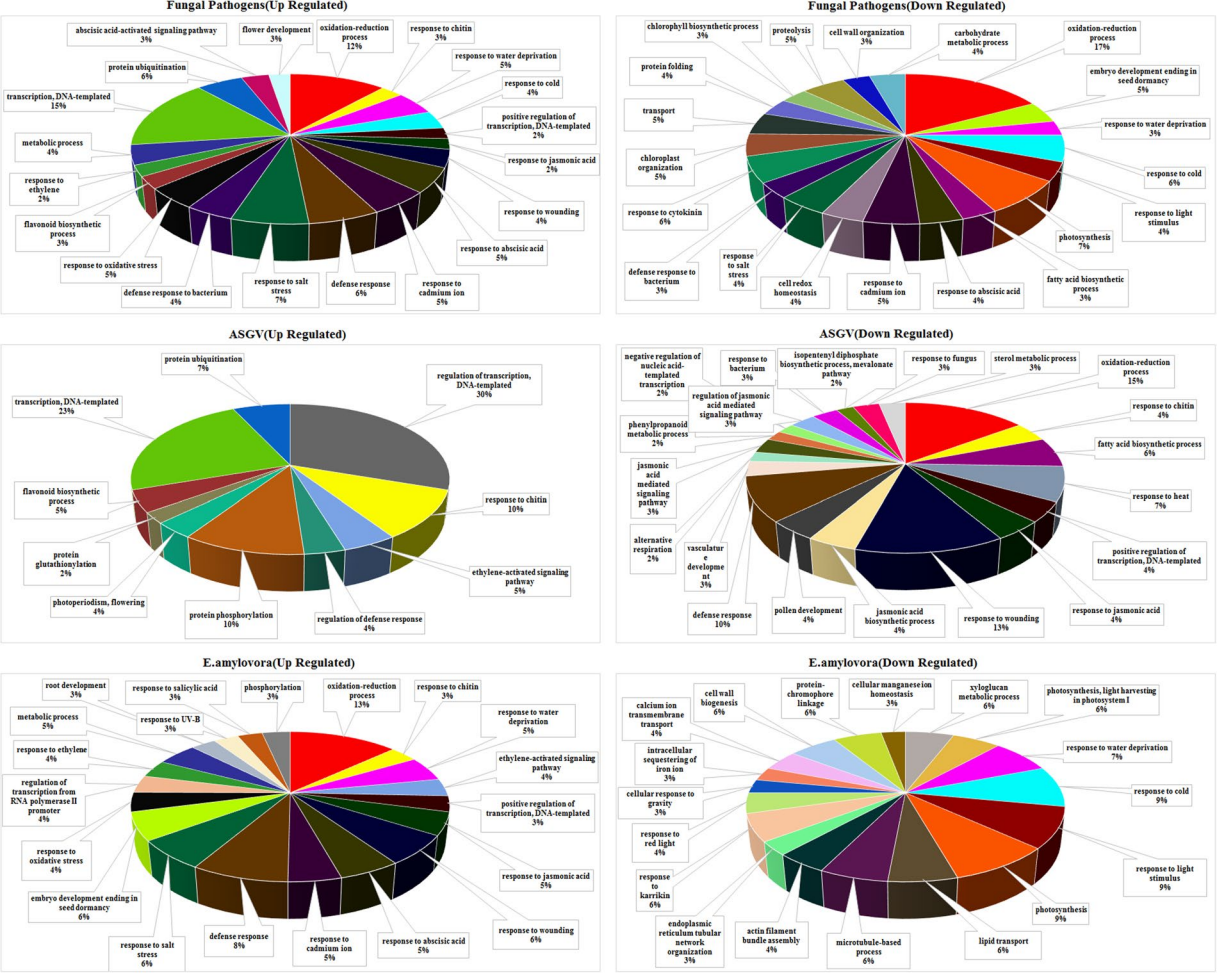
Distribution of gene-ontologies of biological processes for up- and down-regulated genes in the three type of biotic stresses: Fungal Pathogens, Apple stem grooving virus (ASGV) and Erwinia amylovora (E. amylovora).

Gene Ontology enrichment analysis was conducted to explore other possible functions of the differentially expressed genes in different biotic stress conditions. Pie charts showing the distribution of upregulated and downregulated GO-terms in biological processes for each of the three types of biotic stresses were generated (Fig. 1). It is clear that Apple Stem Grooving Virus (ASGV) upregulated a higher percentage of GO-terms related to transcription regulation, response to chitin and phosphorylation. Percentage of upregulated GO-terms related to ethylene and jasmonic acid defense responses were higher in response to Erwinia amylovora (E. amylovora) than to fungal pathogens. Oxidation- reduction pathways were strongly repressed in response to fungal pathogens. In the enrichment analysis, strong differences were observed between the three types of stresses in relation to repressed GO-terms. While fungal pathogens inhibited hormone-related genes, photosynthesis, responses to abiotic stresses, E. amylovora reduced responses to xyloglucan metabolic process, actin, cellular responses to gravity and lipid transport. Virus infection specifically repressed jasmonic acid-related GO terms, response to bacterium and fungal pathogens.

### Gene set enrichment analysis

**Figure 2:**
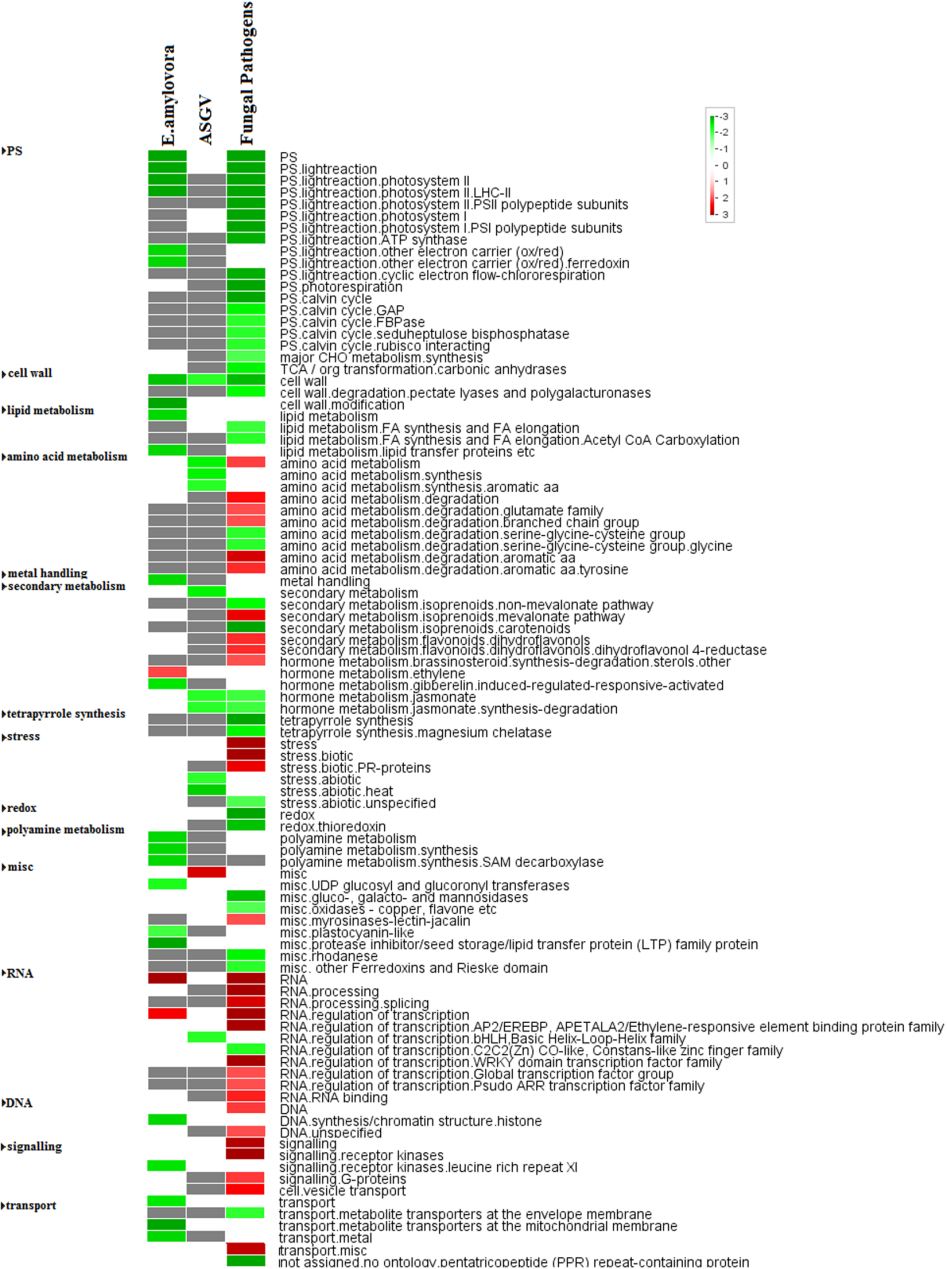
Gene set enrichment analysis of the differentially expressed genes of the three type of stresses: a) Fungal Pathogens b) ASGV and c) E. amylovora. The red color indicates the up-regulated categories and green indicates down-regulated.

Gene enrichment analysis was carried out using pageman^9^ to identify any relation between the expression and function of differentially expressed genes in different biotic stress conditions (Fig. 2). As expected, E. amylovora and fungal pathogens repressed photosynthesis-related genes such as those involved in photosystem II. Adenylpyrophosphatase (ATPase), photorespiration, calvin cycle and major CHO metabolism were significantly inhibited by fungal pathogens while genes encoding electron carriers were repressed by E. amylovora. Cell wall genes were downregulated in all the three datasets. Fungal pathogens upregulated several gene set categories involved in both primary and secondary metabolism including amino acids (glutamate, aromatic ones), flavonoids and isoprenoid mevalonate pathway. PR-proteins and other stress related proteins were upregulated by fungal pathogens.

Relating to hormones, brassinosteroids were induced by fungal pathogens while E. amylovora enhanced ethylene gene set category. Jasmonate was repressed by fungal and viral infections. Most of the transcription factors were induced by fungal pathogens such as AP2-EREBP, WRKYs and ARRs. Ubiquitine-mediated degradation was mainly upregulated by fungal pathogens. Different gene transport-related categories were repressed by different types of pathogens: transporters in envelope membrane by fungal pathogens while metal transporters were downregulated by E. amylovora. Overall, the gene set enrichment analysis identified additional responses due to various biotic stresses in Malus.

### Metabolism overview

**Figure 3:**
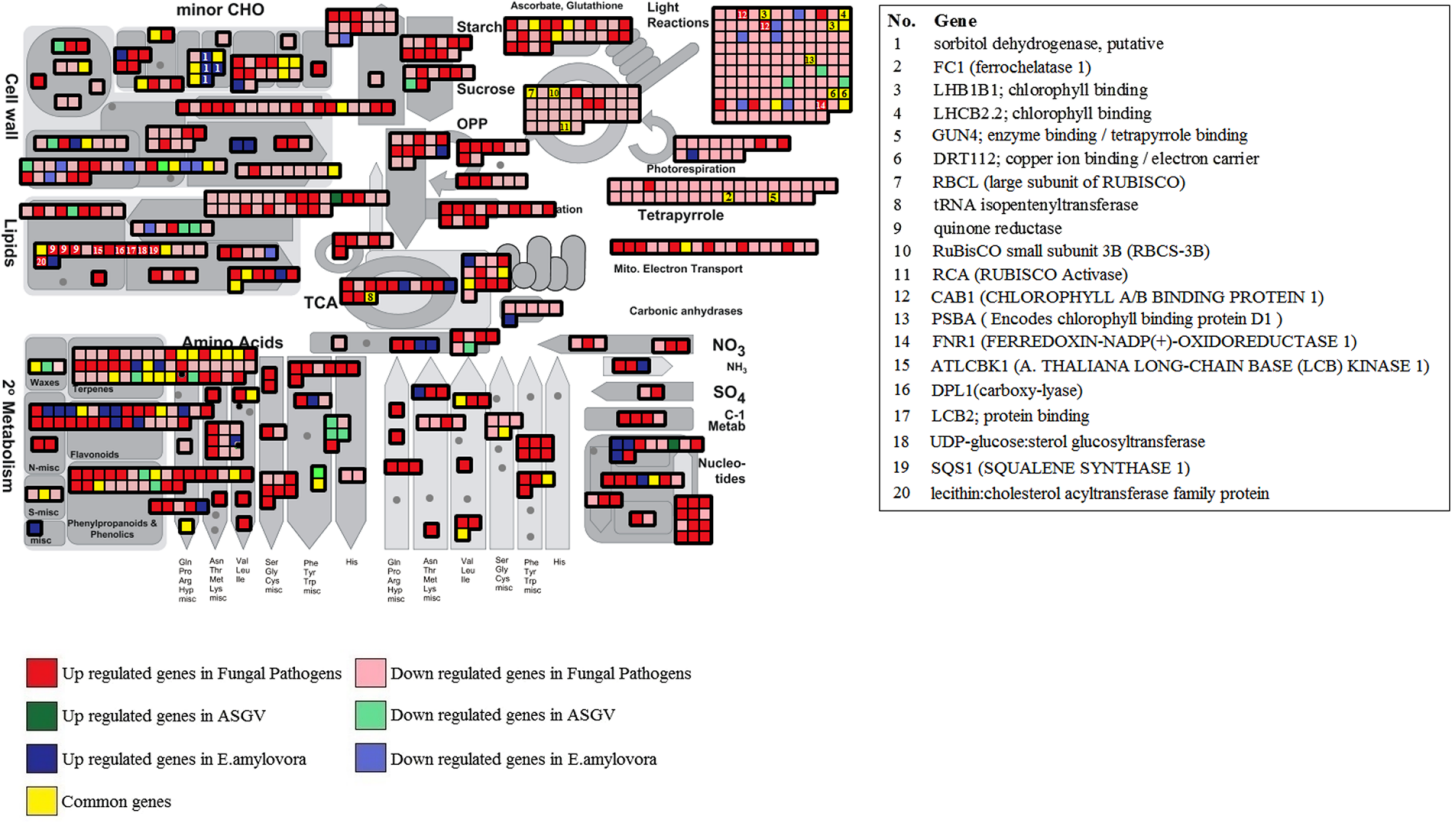
Mapman metabolism overview of differentially expressed genes divided in 7 categories based on their pattern of expression. The important genes were indicated in the figure.

We used Mapman^10^ web-tool to visualize the metabolome changes in Malus X domestica due to biotic stress by using the transcriptomic data of the 7 datasets. Metabolism overview clearly showed the high number of downregulated genes by fungal pathogens involved in light reactions, photorespiration, calvin cycle, photorespiration and tetrapyrrole pathways (Fig. 3, Supplementary Table 8). There were several genes commonly related between at least two of the three types of pathogens such as those genes related to phosynthesis: cholorophyll binding (LHB1B1, LHCB2.2), photorespiration-related genes (FC1, GUN4), large and small subunits of Rubisco Protein (RBCL, RBCS), rubisco activase. Several genes were induced by fungal pathogens involved in TCA cycle, detoxifying mechanisms (ascorbate and glutathione), gluconeogenesis, starch and fermentation, lipid metabolism. On contrast, E.amylovora and viruses repressed genes involved in cell wall modifications. Although three genes were commonly regulated by more than one stress, E. amylovora seems to induce specific expression changes in sugar alcohol metabolism. As far the secondary metabolism concerns, an induction of genes involved in phenylpropanoids and phenolics were mostly induced by fungal pathogens. Ten genes involved in terpene pathways were commonly regulated. Degradation of nucleotides were mostly enhanced by fungal pathogens. In contrast, Mapman^10^ displays large gene expression datasets from different studies in a single metabolic pathway diagram, which help us to easily identify the key genes and its details in different functional categories (Fig. 3).

### Hormone-related pathways

**Figure 4:**
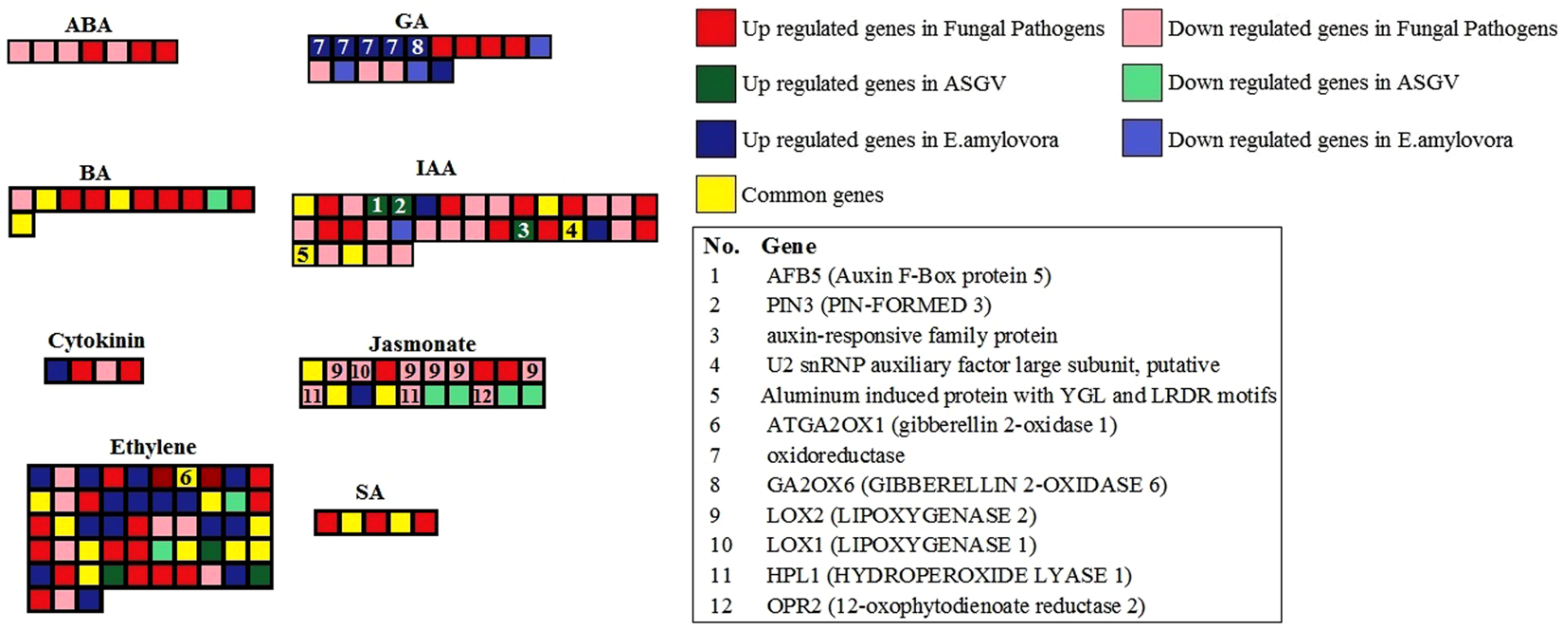
Hormone-related genes affected by biotic stresses in Malus x domestica. The important genes were indicated.

It is very important to study the plant hormonal responses because the signaling pathways of different hormones regulate biotic stress responses antagonistically. We observed that several genes involved in auxin(IAA), benzyl-adenine (BA), ethylene, jasmonate, saliciylic acid (SA) were commonly affected in all types of pathogens. Fungal pathogens upregulated ethylene, benzyl-adenine, salicylic acid while mainly repressed jasmonate-related genes. Erwinia amylovora upregulated ethylene and gibberellin-related (GA) genes while viruses affected some key genes involved in ethylene and auxins. The results suggest that the hormone-related pathways and consequently their crosstalk was profoundly affected by all Malus pathogens (Fig. 4, Supplementary Table 8).

### Detoxifying pathways and secondary metabolism

It is very important to study the underlying detoxification factors and signaling mechanism behind plant defense against biotic stress tolerance. We found that the genes encoding Cytochrome P450 were commonly affected by all three types of pathogens (Supplementary Figure 2, Supplementary Table 8). Fungal pathogens induced UDP Glycosyltransferases, Phosphatases, Nitrilases, Glutathione-S-transferases. In addition to UDP-Glycosyltransferases, viruses mainly enhanced the expressions of Oxidases and Glutathione-S-transferases. Alcohol dehydrogenases, Nitrilases, O-Methyltransferases and Peroxidases genes were upregulated by Erwinia amylovora. Two genes encoding GDSL-lipases were commonly regulated by all the three types of pathogens.

**Figure 5:**
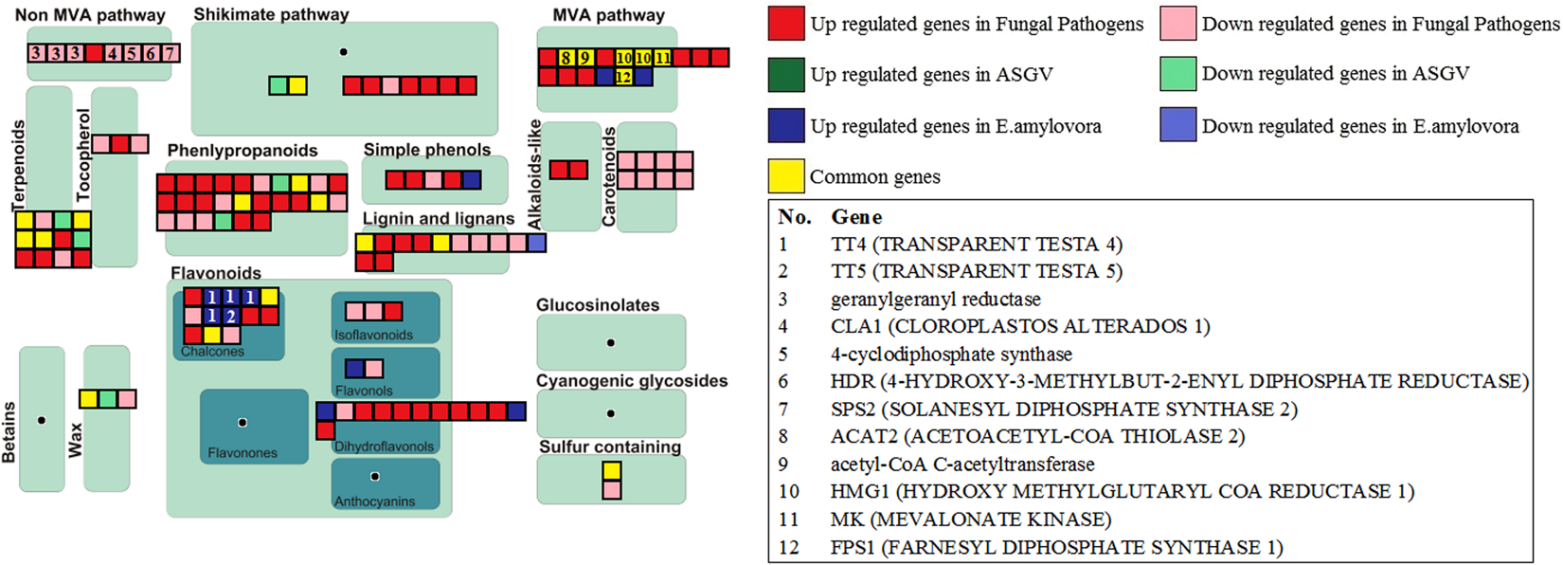
Secondary metabolism genes affected by the three different biotic stresses. The key genes were indicated in the figure.

Clear differences in pattern regulation were observed for fungal pathogens in relation to secondary metabolism gene categories (Fig. 5, Supplementary Table 8). While Phenlypropanoids, Shikimate pathway, Dihydroflavonols, MVA pathway, Simple phenols were mostly induced, Non-MVA pathway, Carotenoids were repressed. On the other hand, Erwinia amylovora mostly upregulated the genes in Chalcones metabolism and MVA pathway. Overall, we observed that the biotic stress influenced the secondary metabolites in Malus X domestica, since these are the compounds which are important for the plant to interact with the environment for adaptation and defense.

### Transcription factors and defense stress-related genes

**Figure 6:**
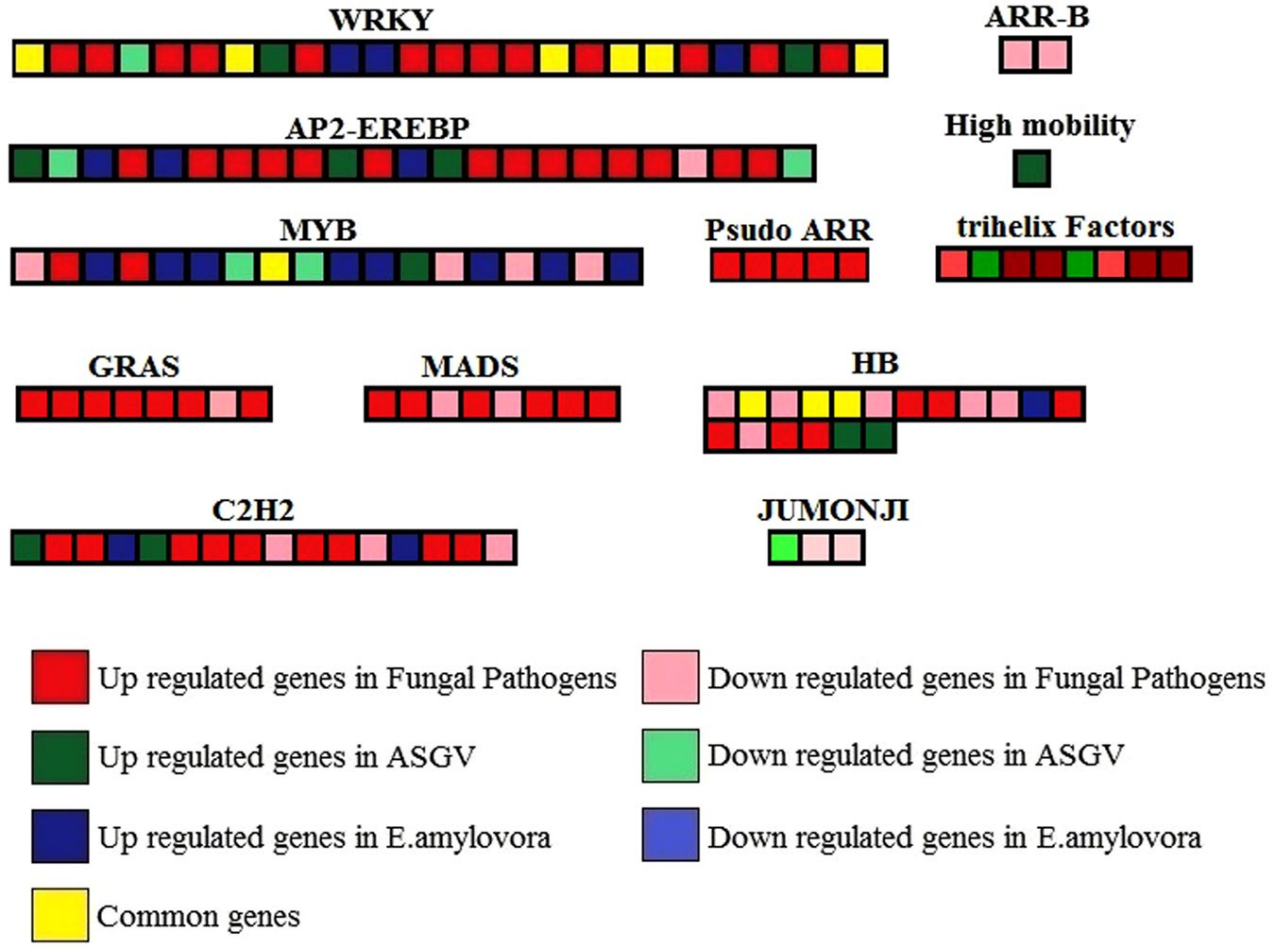
Genes encoding transcription factors and affected by the different categories of biotic stress. The important genes were indicated.

We used Mapman^10^ software to understand the influence of biotic stress in metabolism, hormone regulation, large enzyme families, secondary metabolism and transcription factors. As far as it concerns, transcription factors were drastically affected by the three types of stresses (Fig. 6, Supplementary Table 8). RAP2.3, ERF110, CRF4, four RAP2.4, six AP2 domain-containing transcription factor family proteins, two CEJ1 and one unknown protein in AP2-EREBP were upregulated by fungal pathogens. GRAS factors (SCL1, SCL3, RGA1, SCL13), MADS box (AGL8, AGL24, AGL20, AGL42), C2H2 (MGP, STZ,SEU,STOP1), Psudo ARR (PRR7, PRR5, PRR1) were enhanced by fungal necrotrophic pathogens. Viruses specifically induced some key genes encoding two AP2-EREBPs (AP2, TEM1), two C2H2(RHL41, zinc ion binding), two HB (GL2, BLH3), two trihelix factors (GT2, trihelix DNA-binding protein). Erwinia amylovora upregulated specifically MYB factors (MYB42, MYB15, MYB14, AtMYB111, DNA binding, AtMYB74, MYB33, MYB62) and JUMONJI (cyclin-like F box, jmjC). The complete gene information of the transcription factors were given in Supplementary Table 8.

As expected WRKYs were mostly induced by all 3 kind of biotic attacks (Supplementary Table 2). WRKY11, WRKY32, WRKY33, WRKY35, WRKY40, WRKY6, WRKY65, WRKY69, WRKY70, WRKY72, WRKY75 and TTG2 were induced by at least one of the 5 fungal pathogens. WRKY53, WRKY70 and WRKY35 were enhanced by ASGV. WRKY75, WRKY33 were specifically induced by E. amylovora.

Other genes involved in biotic stress responses were drastically affected by all the three types of stresses. One gene involved in respiratory burst was commonly regulated (Supplementary Figure 3, Supplementary Table 8). Four signaling MLO-like genes were upregulated by fungal pathogens while one was induced by E. amylovora and one was commonly regulated between stresses. In general it is clear that pathogenesis-related proteins were more induced by fungal pathogens than viruses and E. amylovora. Fungi-driven upregulated genes belonged to TIR-NBS-LRR, ATP binding, CC-NBS-LRR, ADR1-L1, RPP1. Four PR-related genes were induced only by E. amylovora while only one disease resistance gene was commonly regulated.

Our results demonstrated that most of the transcription factors and defense stress-related genes were influenced by all types of biotic stresses and also identified the crucial genes response to each type of biotic stress conditions.

### Commonly regulated genes among biotic stresses

It is very important to find the genes which are regulated unique to each type of biotic stress and also commonly regulated by all types of biotic stresses. We found that a total of 322 genes were commonly affected by at least 2 of the 3 types of biotic stresses (Supplementary Table 3, Fig. 3, Supplementary Table 8). These genes represent common responses to stresses and might be helpful to characterize general stress responses in Malus. A great number of these genes were linked with the repression of photosynthesis. Eight genes involved in minor CHO metabolism were affected. Terpenes were affected by all three stresses such as acetyl-coa thiolase2, hydroxyl methylglutaryl coa reductase 1 (HMG1), farnesyl diphosphate synthase1, lyase – magnesium ion binding, beta-amyrin. Also MVA pathway was affected by the three stresses as shown by the differential expression of HMG1, MK, FPS1, ACAT2, Acetyl-CoA (Fig. 3, Supplementary Table 8). Four WRKYs were commonly regulated by at least 2 of 3 types of pathogens: WRKY40, WRKY75, WRKY33, WRKY35 and WRKY70 (Supplementary Table 2). This is a very significant information to identify the targets for genetic modification to improve plant resistance to multiple biotic stresses.

### Inferred protein-protein interaction network analysis

**Figure 7:**
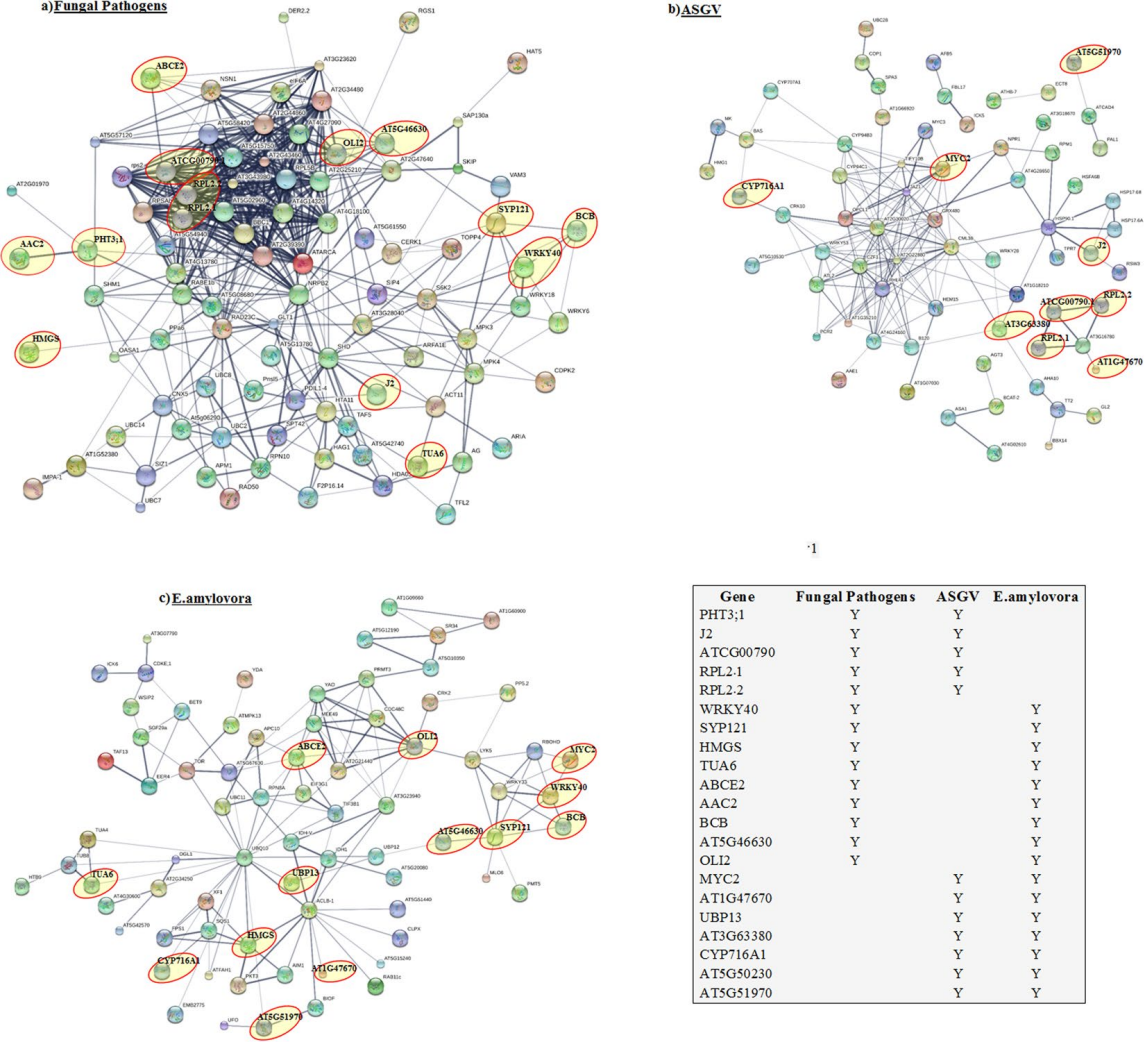
Inferred protein-protein interaction network based on Arabidopsis knowledgebase for the pathogen-regulated genes encoding highly interactive proteins. The common genes (present in more than one in the three groups (1) Fungal Pathogens, (2) ASGV and (3) E. amylovora) are highlighted in the red oval shape and also indicated in the given table. **Y**-indicates that the gene is present in the group.

To understand the degree of conservation in the protein-protein interaction in Malus X domestica in different biotic stress conditions, we visualized the network of the 100 top highly interactive proteins for each of the three types of biotic stresses. The list of top 20 highly interactive proteins of each groups were given in Supplementary Table 4. Arabidopsis orthologs of the Malus pathogen-regulated genes were determined and the protein-protein interactions were determined basing a combined file of inferred and validated interactions^11^ (Supplementary Table 5). The network was visualized using STRING^12^ software (Version 10.0). A highly dense core of 22–23 highly interactive proteins was observed on the top of the network of fungal pathogens (Fig. 7a). Some well-known proteins players in biotic stress responses were noticed such as WRKY40, WRKY18 and WRKY6. These proteins were connected with MPK3 and MPK4. Relating to ASGV infection, MYC2, GRX480, JAZ1, PCL1, RHL41, WRKY53 were hub proteins of this network. Of them only MYC2 was also present in other biotic stresses (E. amylovora). Four HSPs were significantly regulated by virus infections and strictly connected each other (Fig. 7b). A small network composed of four interactive proteins such as RPL2.1, RPL2.2, ATCG00790.1 and AT1G47670 in ASGV was overlapping with fungal pathogen network. NPR1 was present in the virus-affected Protein-Protein Interaction (PPI) network together with the well-known interactive protein GRX480. E. amylovora affected a network connected with five highly interactive genes such as MYC2, WRKY40, WRKY33, BCB, SYP121 and AT5G46630 (Fig. 7c). Some key highly interactive ubiquitin proteins were observed in the network such as UBQ10. Sixteen E. amylovora proteins regulated at transcriptional level were commonly present also in at least one of the other stresses. The protein-protein interaction network analysis helped to minimize the complexity in understanding physical interaction between proteins due to different biotic stresses.

## Discussion

The need of more meta-analysis of transcriptomic studies is due to several reasons. First, transcript amounts are highly affected by changing environmental and developmental conditions. Secondly, field studies are usually conducted only in one season leading to unreliable results affected by a high number of environmental disturbing factors. Third, few replicates (frequently only three) are usually considered due to the high costs of “omic” analysis. Fourthly, transcriptomic studies should be integrated with proteomics and metabolomics in order to clarify post-transcriptional and post-transductional regulation mechanisms. Finally the identification of commonalities between similar independent studies will identify which gene are more strongly associated with the subject of the study and focus the functional analysis only on those common findings^13^.

Here we showed data of a meta-analysis of 7 published transcriptomic articles dealing with biotic stress responses in Malus x domestica. At the moment, in Scopus database, there are 5 articles related to fungal pathogens, one related to virus and one to bacteria (E. amylovora).

The significant downregulation of light reactions in response to both fungal pathogens and E. amylovora was expected due to the symptomatic stages reported in 6 analyzed articles^1–4,14,15^. These evidences have been previously reported as a typical response clearly shown not only at phenotypic but also evident in previous gene set enrichment analysis^16,17^. Carbohydrate metabolism has been frequently shown as a key pathway affected by biotic stresses responses in plants^18^. Growing tissues may be seen as a collection of sinks of carbohydrate attracting photosynthates produced by leaves. A correct mechanism of source-sink relationship allows carbon allocation during abiotic and biotic stress consequently improves plant performance in harsh environments^19^. The source-sink disruption has been linked with the early pathogenic mechanisms of diseases in plants^16,17^. Indeed we believe that the dysregulation of this pathway at transcriptomic level may be associated with a general plant stress state. This altered transcript condition may be seen by growers as a sort of “alarm bell” to help further monitoring actions and the beginning of management procedures. Sugar alcohols are acyclic polyols produced outside the chloroplast and they are directly linked with stress responses. Although their role in tolerance to stress has been more linked with abiotic than biotic stresses, it has been hypothesized that they may play a key role also in a beneficial modulation of biotic stress responses^20^. Our meta-analysis pointed out how sugar alcohols may be more involved in responses to bacterial pathogens.

The repression of detoxifying genes such as those involved in ascorbate and glutathione-S-transferases mainly observed in response to fungal pathogens is a clear evidence of pathological status. Recently the over-expression of these genes have been linked with an increased tolerance to Huanglongbing disease in citrus^21^. Glutathione S-transferases (GSTs) are proteins encoded by a large family of genes and involved in host defenses against environmental stresses. The transgenic overexpression of a GST in tobacco drives to an increased resistance to Fusarium oxysporum^22^ agreeing with our findings that show a significant upregulation of these genes in responses not only to fungal pathogens but also to ASGV and *E. amylovora*. Although the role of glutathione in functioning as protectors during plant abiotic stresses still remains to be unclear, a recent work highlighted its importance as signal of hormones and other protecting molecules^23^.

Interestingly, we observed that polyamine metabolism was repressed by *E. amylovora* but not by the five fungal pathogens. It is well-known that these molecules are increasingly accumulated in response to stresses as well as transcript abundance of genes involved in their biosynthesis are generally upregulated. In addition, the transgenic overexpression of these genes enhanced resistance to stresses and several studies showed their key role in the modulation of intra-cellular levels of reactive oxygen species^24^. It is intriguing why polyamine metabolism resulted to be repressed by E. amylovora. It remains to be clarified if they might play a key role in the pathogenetic mechanisms of fire blight in Malus.

Interestingly *E*. *amylovora* significantly repressed the category of Lipid transport and this may promote the occurrence of the progression of the symptomatology. These proteins are specific pathogenesis-related proteins involved in plant defense responses^25^. These proteins are involved in the inhibition of pathogen growth^26^.

A predominant number of AP2/EREBP TFs were upregulated in comparison to the downregulated ones. The five fungal pathogens significantly upregulated the APETALA2/ethylene-responsive element binding protein (AP2/EREBP) transcription factors while three of them were induced by the ASGV and three by E. amylovora. Only one was commonly regulated between two of the three pathogens. This evidence leads us to speculate that the three kind of pathogens induce exclusive signaling to activate specific immune responses. Their key role in signal transduction of plant hormones is well-known^27^. A comprehensive analysis have been conducted in *V. fordii* and *V. montana* and showed how different members may be up- or down-regulated depending on the two species in response to *Fusarium oxysporum*^28^. RAV2 was one of these proteins specifically induced by E. amylovora. The constitutive overexpression of this gene in tomato enhanced ERF5 and PR5 genes increasing the tolerance to bacterial wilt^29^. Previous works suggest that RAV1 may work as a transcriptional activator inducing resistance to bacterial infection^30^. Taken together, these findings lead to speculate that RAV genes may be more involved in bacterial defense than fungal and virus pathogens.

GRAS transcription factors are involved in plant disease resistance^31^. Interestingly we noticed five SCL genes that were upregulated by fungal pathogens but not by the other two types of pathogens although RGA1 was commonly regulated. GRAS proteins are repressors of gibberellin signaling due to the presence of the N-terminal region amino acid sequence DELLA and are considered DELLA proteins^31^. Indeed, the downregulation of three gibberellin-responsive genes observed by the fungal pathogens agree with the upregulation of GRAS proteins.

MYB proteins present a repeated numbers of MYB domains that allow them to bind DNA. They are commonly expressed in plants and regulated by diverse environmental factors. Their role in ABA-response is well-recognized^32^. Interestingly, 8 MYB proteins were induced only by E. amylovora and not by the other pathogens including MYB62 and MYB15. Only MYB6 was commonly regulated between 2 of the 3 kind of pathogens. MYB62 has been linked with phosphate starvation^33^ while MYB15 was induced by wound and insect herbivores responses^34^. From our analysis it seems that, at least in Malus, MYBs are more linked with E. amylovora than fungal pathogens and AGSV.

Finally, another important category of transcription factors affected by all three types of pathogens was WRKYs. They are well-known for their key role in response to many different environmental stresses^35^. Thirteen WRKYs were upregulated in response to fungal pathogens, 3 in response to E. amylovora and 2 to viral infection. The WRKYs that were commonly regulated between all the biotic stresses are interesting because of their important role in the modulation of the hormonal cross-talk in response to pathogens. Six of them were commonly regulated in at least two types of biotic stress including WRKY70 and WRKY40. Since this gene was highly expressed in plants treated with ethylene (ET) and salicylic acid (SA) while it was repressed in response to methyl jasmonate (MeJA), its key role in SA-JA crosstalk has been hypothesized^35^. This protein showed to have repressive effect on SA-mediated defense while it contributes to stimulate JA-mediated responses. WRKY33 was upregulated by all three types of biotic stress. This gene has been linked with bacterial infections^16,21,36^ and it is upregulated by Trichoderma, a fungal genus that stimulates plant and root growth and nutrient uptake^37^. WRKY53 was upregulated only by ASGV. It has been shown that two key genes involved in biotic stress responses, a Ser/Thr receptor kinase ORK10/LRK10 and an apoplastic peroxidase were targeted by WRKY53^38^. WRKY75 was enhanced by E. amylovora. Interestingly its transgenic overexpression allowed to improve resistance to Sclerotinia sclerotiorum^39^.

Interestingly a clear upregulation of ethylene-related genes were observed more in response to E. amylovora than to the other two biotic stresses. In total, nine 2OG-Fe(II) oxygenases were upregulated by bacterial infection. Considering the total number of *E. amylovora*-regulated genes, ethylene-related category was highly represented in gene set enrichment analysis. The upregulation of ERF1 by E. amylovora was expected since this gene has been linked with the enhancement of JA-responsive genes through ORA59^40^. Data related to Jasmonic acid responsive genes were contrasting in response to E. amylovora. While an allene oxydase synthase gene was induced, two genes (an allene oxide synthase 2 and 4) were repressed. GASA4 was repressed by E. amylovora. This gene is part of a family of GA-inducible and ABA-repressible genes. It is generally induced by hormones involved in growth development while it is repressed by stress-related hormones (ABA, JA, and SA), implying its key role in hormone crosstalk^41^. Fungal pathogens predominantly upregulated the genes involved in ethylene, brassinosteroids and salicylic acid while jasmonic acid responses were mostly repressed. This was expected since Venturia inaequalis, one of the studied fungal pathogen, is considered a hemi-biotrophic pathogen. Relating to responses to ASGV, an upregulation of two auxin responsive genes, AFB3 and PIN3 were observed. PIN3 is an auxin transporter that plays a key role in root growth and lateral architecture mediated in the hypocotyl^42^. Although the role of this gene in pathogen defense responses has to be elucidated, it may be somewhat affected since exogenous SA showed that mostly repressed Pin-formed (PIN) genes^43^.

Different categories of genes involved in secondary metabolism were selectively regulated by apple stem grooving virus, fungal pathogens and E. amylovora. While fungal pathogens upregulated shikimate pathways, MVA and phenylpropanoids, E. amylovora clearly induced genes involved in chalcones. On contrast, non-MVA was clearly repressed by fungal pathogens. MVA pathway is responsible for terpenoid biosynthesis and was commonly affected by the studied pathogens as shown by the significant regulation of 6 genes. Terpenoids comprise a series of metabolites with peculiar protection roles to biotic attacks. Several volatile sesquiterpenes are important chemical signals for the activation of plant defence mechanisms in response to biotic stresses. The wide range of different terpenoids present in plants, implied that they should have posed an important role in plant evolution in response to different ecological plant interactions with both biotic and abiotic aspects.

The expression of genes involved in phenylpropanoid metabolism was clearly induced by fungal pathogens. They have important protective roles towards both biotic and abiotic stresses and they are regulated by MYB transcription factors^44^. Increased amount of phenylpropanoid transcripts were also associated with Citrus responses to Huanglongbing disease^45^. Secondary metabolism genes including chalcone isomerases were upregulated by Marssonina coronaria. Three genes were commonly regulated between stresses while 5 naringenin-chalcone synthase genes were upregulated by E. amylovora. Both chalcones and dihydrofavonols were upregulated by the fungal pathogens and E. amylovora. These compounds belonged to flavonoids, an important class of secondary metabolism compounds with protecting functions against fungal infection. They are categorized into two groups: constitutively expressed and stimulated. The first category are usually maintained in particular locations and used as signals when pathogen attacks occur^46^ while the second comprises genes induced during plant-pathogen interactions. These compounds exercise a protection role thank to their antioxidant capabilities, cross-linking and inhibition of microbial proteins such as cell wall degradation enzymes, metal chelation as well as physical barrier against pathogens^47,48^. Interestingly carotenoid genes were repressed by fungal pathogens. Their protection role in plant resistance to biotic stresses was shown in mutant experiments that demonstrated their important role in ROS detoxyfication under stress conditions^49^. Results of this meta-analysis suggest that the repression of these genes in response to apple fungal pathogens might provoke negative effects on the progression of the disease.

Relating to fungal pathogens, a core of 22–23 highly proteins were clearly observed by the observation of the overall network. Among these proteins, there were RPL5B, BBC1, ATARCA. Three WRKYs, WRKY6, WRKY18 and WRKY40 were significantly regulated by fungal pathogens in Malus. WRKY18 and WRKY40 proteins formed complexes and presented DNA binding properties. These WRKYs are involved in pathogen-induced HR linked with the induction of salicylic acid (SA)–mediated immune responses causing the progression of the systemic acquired resistance (SAR). WRKY18 and WRKY40 have common sequences with more than 60% identical amino acids^50^. WRKY18, WRKY40, and WRKY60 showed to negatively affect resistance to hemibiotrophic pathogens^50^. In addition, different WRKYs seems to have contrasting effects on response to Pseudomonas syringae and to B. cinerea. Indeed, these three WRKY proteins may be negative regulators of the SA-dependent pathways while they induce JA-mediated pathways. A mitogen-activated protein kinase 4 (MPK4), inhibitor of SA-dependent resistance^51^, was shown to interact with WRKY25 and WRKY33 implying that their role in response to necrotrophs might be complex^52^. It seems that resistance to the fungal pathogens in Malus was associated with specific expression levels of these three WRKY genes. Hypersensitive reaction is important for the virulence of the fungal pathogen B. cinerea^53^. SA and ET upregulates signalling pathways antagonistic to each other, but both of them enhance pathogen-induced cell death^54^. Therefore, it is possible that WRKY genes cause activation or suppression of diverse signalling mechanisms in response to necrotrophic pathogens promoting virulence. It is worthy to notice that cooperative bonds with different WRKY proteins might regulate their activity as transcription factors. Thirteen proteins interactive at protein-protein level were commonly regulated with the other two biotic stresses and may be considered a general plant stress state.

MYC2 was differentially regulated by both ASGV and E. amylovora. MYC2 is considered as a key regulating protein of JA signalling in Arabidopsis^55^ since it interacts with JASMONATE ZIM-domain proteins. Transgenic increased expression of *OsMYC2* stimulated the expression of early JA-responsive genes, inducing bacterial blight resistance through JA-hypersensitive reaction^56^. Interestingly we observed a protein-protein interaction network shared with the other biotic stresses and consisting of key proteins such as MYC2, WRKY40, BCB, SYP121. The PPI network showed that WRKY33, WRKY40 and MYC2 were strictly connected and significantly regulated by E. amylovora. The expression of WRKY40 was enhanced in response to wounding and infections of Ralstonia solanacearum. This gene was regulated by salicylic acid, methyl jasmonate, ethylene^57^. MYC2 interacts with NPR1 that was also significantly affected by ASGV. This gene was upregulated by SA^58^ and its subcellular localization was modulated by redox changes caused by salicylic acid^59^. It binds directly to salicylic acid, releasing its transactivation domain and it is regulated by proteasome-mediated turnover^60^.

Interestingly, ASGV modulated the expression of heat shock proteins such as HSP90.1, HSP176B, HSP17A and HSFA6B. The PPI network showed that these proteins were strictly interacting with each other. Heat shock proteins were frequently induced in cells of all organisms in response to heat^61^. Their function is to protect protein folding since they are able to reduce protein misfolding due to all kind of stresses. HSP90 was involved in signal transduction of plant responses through the interaction of a salicylic acid-induced protein kinase. HSP90 affects defense responses against pathogens through specific interactions with other genes, working as a scaffold in protein complexes involved in signal transduction^62^. Along with transcriptomics analysis, the protein-protein interaction network analysis help us to visualize and identify the key node proteins which are affected pathogen infections. Thus the meta-analysis plays a major role in identifying potential biomarkers for different biotic stress conditions in plants by comparing different omic data sets pertaining to a specific functional context^36^.

## Methods

### Search strategy of published study identification and selection for meta-analysis

We identified all published transcriptomic studies in Malus x domestica in Scopus and PubMed before March 2017, using the combination of keywords ‘Transcriptomics” and “malus” or ‘Transcriptomics” and “apple” in computer-based searches. The RNA-Seq studies pertaining to biotic stress on Malus x domestica were selected and classified into three groups a) Fungal pathogens^1–3,14,15^, b) ASGV^5^ and c) E.amylovora^4^ based on the pathogen type. The list of differentially regulated genes, obtained from the selected seven published transcriptomic articles in Malus x domestica were given in Table 1. Only genes reported in the main text and supplementary files of these articles were considered in this meta-analysis.

**Table 1:** Transcriptomic studies dealing with biotic stress responses in Malus x domestica used for meta-analysis.

Article	Objective	Pathogen Species	Pathogen	Tissue	Sample Information			Group
					Total	Up	Down	
Gusberti, M. *et al*.^14^	Resistance to Venturia	Venturia inaequalis	Fungi	Leaf	212	112	100	Fungal Pathogen
Yin, Z. Y. *et al*.^1^	Resistance to Valsa mali	Valsa mali	Fungi	Twig	29	14	15	
Xu, J. H. *et al*.^15^	Response to Marssonina coronaria inoculation	Marssoninacoronaria	Fungi	Leaf	90	58	32	
Zhu, L. M. *et al*.^2^	Response to Alternaria alternata	Alternaria alternate	Fungi	Leaf	3854	2108	1746	
Shin, S. B. *et al*.^3^	Response to Pythium ultimum	Pythium ultimum	Fungi	Root	82	63	19	
Chen, S. Y. *et al*.^5^	Apple stem grooving virus	Apple stem grooving virus	Virus	Shoot	320	184	136	ASGV
Kamber, T. *et al*.^4^	Responses to Erwinia amylovora	Erwinia amylovora	Bacteria	Flower	823	640	183	E. amylovora

Number of up-regulated and down-regulated genes were indicated for each studies.

### Extraction and annotation of differentially expressed genes

The up and down regulated genes and the fold change information were extracted from the supplementary tables of the articles. We selected only the genes having fold change and p-value cutoffs (log_2_ FC > 1 or log_2_ FC < −1; p-value < 0.05), in order to strengthen the accuracy of the analysis and normalization; except one article where the fold change is not given^1^. All the Malus x domestica gene ids were based on the Phytozome database (https://phytozome.jgi.doe.gov/pz/portal.html) and were mapped to the corresponding Arabidopsis id (Supplementary Table 5), using the annotation file downloaded from Phytozome. The data extraction and mapping were done by in-house Perl scripts. We merged the 5 fungi datasets in one single file in order to analyze the entire list of fungal pathogen-regulated genes in Malus x domestica. This operation was not needed for viral and bacterial responses since only one transcriptomic study was available for both types of pathogens.

### Gene enrichment analysis and functional analysis

The metabolic overview, hormone regulation, large enzyme families, transcription factors and biotic stress gene categories of the three groups were visualized using MapMan with the Malus x domestica mapping file (Mdomestica_196.txt)(http://mapman.gabipd.org/). The PageMan analysis plugin of MapMan was used to visualize differences among metabolic pathways using Wilcoxon tests, no correction, and an over-representation analysis (ORA) cutoff value of 3.

Gene ontology enrichment analysis was performed using the Database for Annotation, Visualization and Integrated Discovery (DAVID) Web server (https://david.ncifcrf.gov/)^63^, based on the homologous TAIR IDs (Supplementary Table 6). The gene ontology information of each groups were extracted from the DAVID results using in-house Perl script and the top 20 hits were given in Supplementary Table 6 according to biological process, cellular component and molecular function.

### Protein-protein interaction network

The protein-protein interaction network (PPI) information based on both experiment (ppi(exp).v5.03) and integrated prediction based (ppi(pred).v5.03) were downloaded from AtPID (Arabidopsis thaliana Protein Interactome Database; http://www.megabionet.org/atpid/webfile/)^11^. The complete PPI information of the genes of all three groups were extracted and given in the Supplementary Table 5. The top 100 genes for each groups were selected based on the PPI count and were considered for the PPI network analysis. PPI network was constructed based on the protein interaction information retrieved from STRING (Search Tool for the Retrieval of Interacting Genes/Proteins, http://string-db.org/)^12^, an online protein-protein interaction database curated from literature and predicted associations from systemic genome comparisons.

## Conclusions

Comparisons of transcriptomic datasets obtained to study different biotic stress in the same crop allows identifying which genes are specifically involved in disease resistance and which may be associated with general plant stress conditions. Meta-analyses allow increase in reliability of transcriptomic data, reducing environmental variability due to a low number of biological replicates and repeated experiments. In this work, the meta-analysis conducted in Malus, highlights the role of WRKYs in the molecular response to biotic stresses at both transcript and protein-protein interaction levels. Although WRKY40 was involved response to both fungal pathogens and E. amylovora, its interaction with other different WRKY may induce specific responses. In response to fungal pathogens, WRKY interacted with two other pathogen-regulated WRKY6 and WRKY18 while in response to E. amylovora it interacts with WRKY33. Specific hormones were differentially affected between the three types of stresses and drives to specific defense responses. Future studies in other crops investigating similar diseases will allow validate these findings and identify resistance mechanisms in gene regulatory networks of plant-microbe interactions.

## Electronic supplementary material

Supplemental Information

Table S8
