# Supplementary material for: Transcriptomic responses to biotic stresses in Malus x domestica: a meta-analysis study
Source: Sci Rep. 2018 Jan 31;8:1970. doi: 10.1038/s41598-018-19348-4 (PMC5792587; doi:10.1038/s41598-018-19348-4)
Supplement: Supplementary file 1 — Supplemental Information [file 41598_2018_19348_MOESM1_ESM.pdf]

Supplementary information

Transcriptomic responses to biotic stresses in Malus x domestica: a meta-analysis study

Bipin Balan[1], Franco Marra[1], Tiziano Caruso[1], Federico Martinelli[1]

[1]Dipartimento di Scienze Agrarie e Forestali, Università degli Studi di Palermo, Palermo, Italy

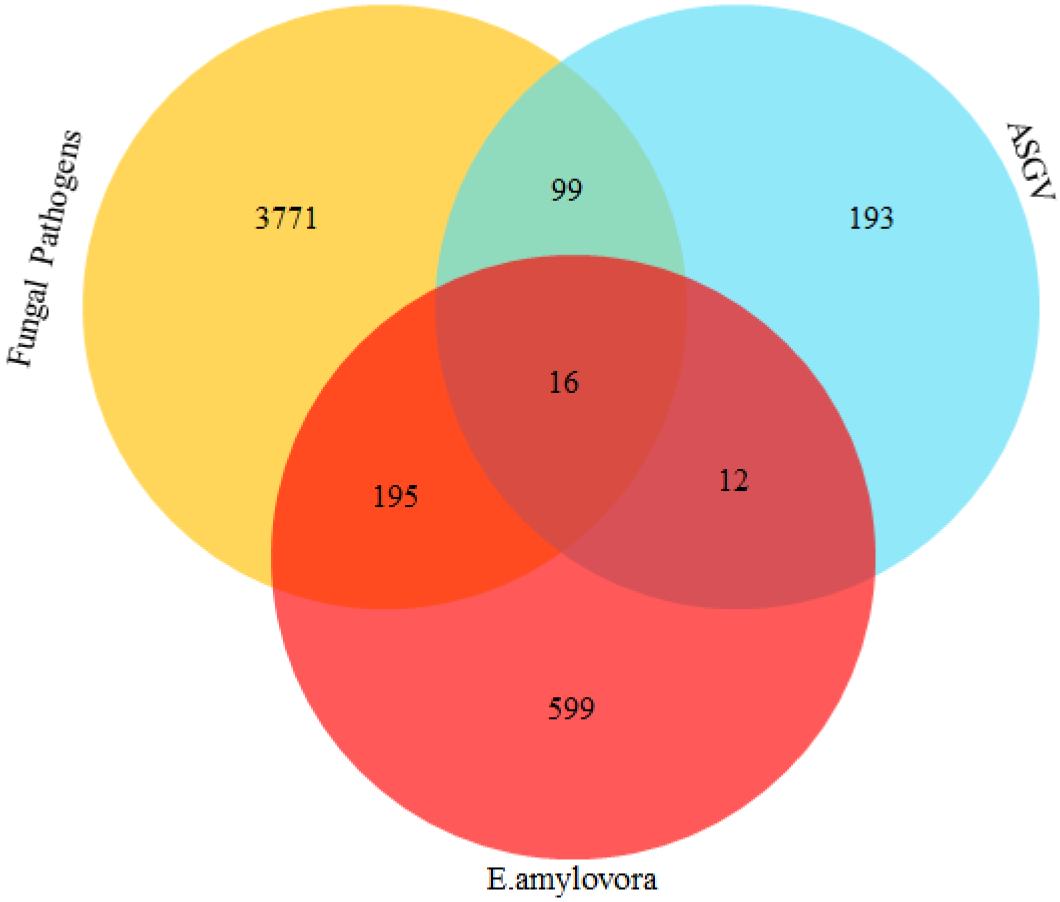

Figure S1. Venn diagram showing the number of specifically and commonly regulated genes for each biotic stresses: Fungal Pathogens, Apple stem grooving virus (ASGV) and Erwinia amylovora (E. amylovora).

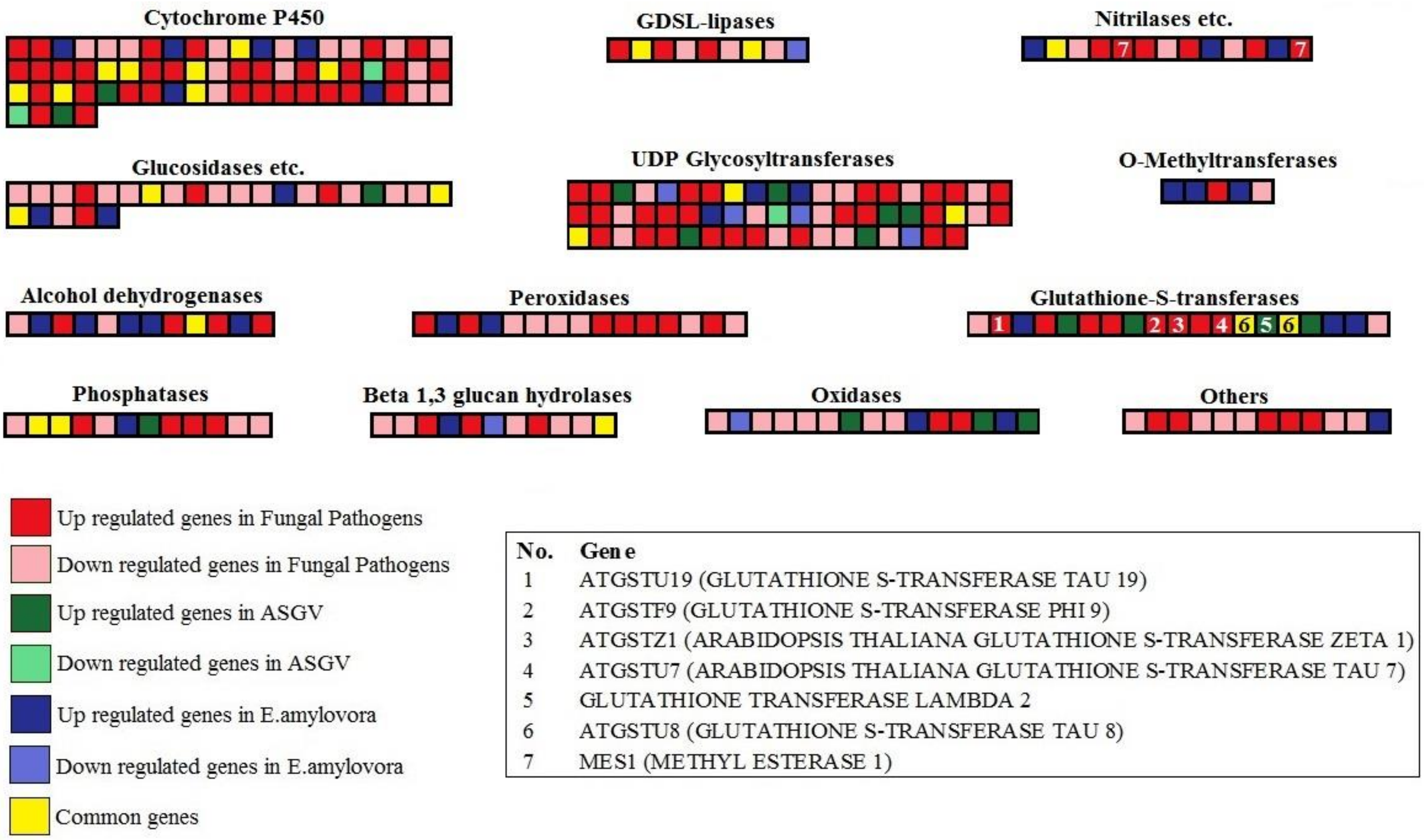

No.	Gene
1	ATGSTU19 (GLUTATHIONE S-TRANSFERASE TAU 19)
2	ATGSTF9 (GLUTATHIONE S-TRANSFERASE PHI 9)
3	ATGSTZ1 (ARABIDOPSIS THALIANA GLUTATHIONE S-TRANSFERASE ZETA 1)
4	ATGSTU7 (ARABIDOPSIS THALIANA GLUTATHIONE S-TRANSFERASE TAU 7)
5	GLUTATHIONE TRANSFERASE LAMBDA 2
6	ATGSTU8 (GLUTATHIONE S-TRANSFERASE TAU 8)
7	MES1 (METHYL ESTERASE 1)

Figure S2. Genes involved in detoxifying pathways and encoding enzymes belonging to different large families. The key genes explained in the manuscript were highlighted in the figure.

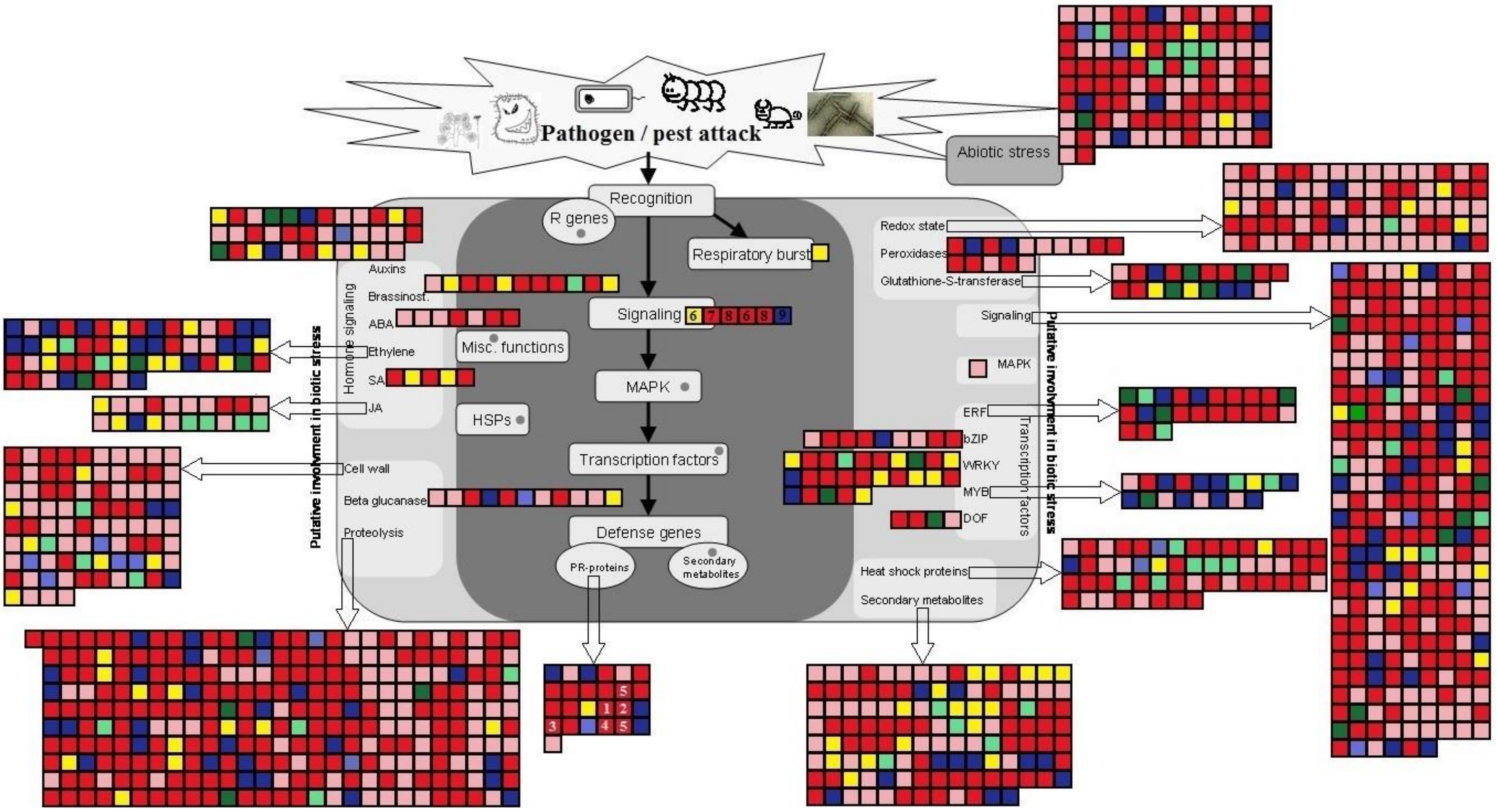

Up regulated genes in Fungal Pathogens
Down regulated genes in Fungal Pathogens
Up regulated genes in ASGV
Down regulated genes in ASGV
Up regulated genes in E.amylovora
Down regulated genes in E.amylovora
Common genes

No.	Gene
1	disease resistance protein (TIR class), putative
2	disease resistance protein (CC-NBS-LRR class), putative
3	ADR1-L1 (ADR1-like 1); ATP binding / protein binding
4	RPP1 (recognition of peronospora parasitica 1); LRR domain binding
5	ATP binding / protein binding / transmembrane receptor
6	MLO6 (MILDEW RESISTANCE LOCUS O 6); calmodulin binding
7	MLO3 (MILDEW RESISTANCE LOCUS O 3)
8	MLO1; calmodulin binding
9	MLO12 (MILDEW RESISTANCE LOCUS O 12)

Figure S3. Biotic stress mapman overview showing genes differentially expressed in the 7 transcriptomic articles and divided in three types of stresses and 7 categories depending on their trend of expression. The key genes explained in the manuscript were indicated in the figure.
